# The Photoperiod: Handling and Causing Stress in Plants

**DOI:** 10.3389/fpls.2021.781988

**Published:** 2022-01-25

**Authors:** Venja M. Roeber, Thomas Schmülling, Anne Cortleven

**Affiliations:** Dahlem Centre of Plant Sciences (D), Institute of Biology/Applied Genetics, Freie Universität Berlin, Berlin, Germany

**Keywords:** biotic stress, cold stress, drought stress, GIGANTEA, osmotic stress, photoperiod, photoperiod stress

## Abstract

The photoperiod, which is the length of the light period in the diurnal cycle of 24 h, is an important environmental signal. Plants have evolved sensitive mechanisms to measure the length of the photoperiod. Photoperiod sensing enables plants to synchronize developmental processes, such as the onset of flowering, with a specific time of the year, and enables them to alleviate the impact of environmental stresses occurring at the same time every year. During the last years, the importance of the photoperiod for plant responses to abiotic and biotic stresses has received increasing attention. In this review, we summarize the current knowledge on the signaling pathways involved in the photoperiod-dependent regulation of responses to abiotic (freezing, drought, osmotic stress) and biotic stresses. A central role of GIGANTEA (GI), which is a key player in the regulation of photoperiod-dependent flowering, in stress responses is highlighted. Special attention is paid to the role of the photoperiod in regulating the redox state of plants. Furthermore, an update on photoperiod stress, which is caused by sudden alterations in the photoperiod, is given. Finally, we will review and discuss the possible use of photoperiod-induced stress as a sustainable resource to enhance plant resistance to biotic stress in horticulture.

## Introduction

Eukaryotes, including plants, adapt numerous life processes to regular rhythms of light and darkness. Light and dark periods regularly alternate in a daily cycle of approximately 24 h due to the rotation of the Earth around its own axis. The duration of the light period during this 24 h day-night cycle determines the photoperiod, which varies with the season and latitude (Jackson, 2009). Plants synchronize their physiological decisions with the correct time of the year to maximize growth and to produce offspring (Casal et al., 2004). Thus, sensing of and responding to the photoperiod are important plant functions to adapt to their environment.

Among the most prominent plant responses influenced by the photoperiod are the regulation of flowering time (Carré, 2001; Song et al., 2015), tuberization (Sarkar, 2010), bud setting, and dormancy (Jackson, 2009; Singh et al., 2017). In annual plants, senescence is adjusted by the photoperiod (Serrano-Bueno et al., 2021) and in perennial plants like trees, the growth cessation is influenced by season-dependent photoperiods (Singh et al., 2017). In temperate climate zones but also in tropical regions, the photoperiod is the dominant environmental factor controlling the onset and end of the seasonal growing (Adole et al., 2019). Scent emission from flowers is also under the control of the photoperiod (Hendel-Rahmanim et al., 2007) to mention just a few examples of photoperiod-regulated developmental processes in plants.

Based on their flowering response to the photoperiod, plants can be classified into three groups: short-day, long-day, and day-neutral plants. This classification is based on the critical day length (CDL), which determines the ability of plants to respond to the photoperiod. Short-day-grown plants flower when the photoperiod is shorter than the CDL, while long-day plants flower only, when the photoperiod is longer than the CDL. Day-neutral plants do not respond to the photoperiod (Jackson, 2009). Besides the CDL, the plant developmental phase determines the ability to sense and subsequently respond to photoperiods. The flowering response of *Arabidopsis* plants is insensitive to photoperiods during their juvenile phase. Entering the adult phase makes *Arabidopsis* sensitive to photoperiods enabling responses to floral inducers (Matsoukas, 2014). Taken together, the synchronization of the photoperiod sensing and intrinsic developmental programs or developmental phases with the seasonal photoperiod is essential for the reproduction and survival of plants.

**FIGURE 1:**
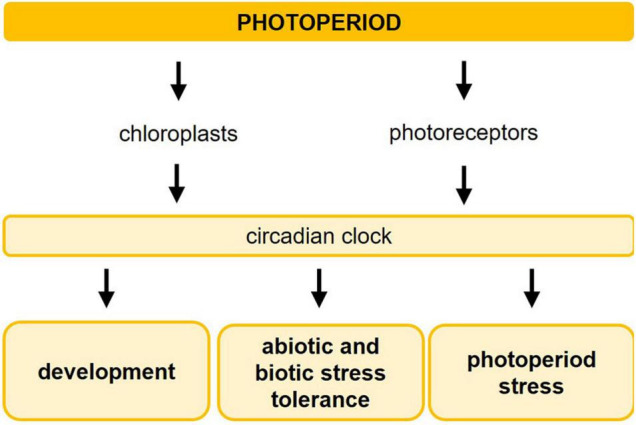
Photoperiod sensing influences development of plants, induces abiotic and biotic stress tolerance, and causes photoperiod stress. The length of the photoperiod is detected by a sensing mechanism consisting of chloroplasts and photoreceptors, which transfer the light information to the circadian clock. The interplay between the photoperiod and the circadian clock regulates developmental processes, such as flowering, tuberization, bud setting, dormancy, and senescence, and improves the plants’ tolerance to abiotic and biotic stresses. A sudden prolongation of the photoperiod results in photoperiod stress.

Photoperiod sensing not only enables plants to synchronize their developmental processes with a specific time of the year but also alleviates the impact of environmental stresses occurring at the same time every year. Recently, the interest in the effect of the photoperiod on the response to abiotic and biotic stresses has grown. For example, the role of shortening of days in cold acclimation to prepare for freezing winter temperatures has been uncovered (Ouellet and Charron, 2013). The photoperiod has also been shown to influence the plants’ resistance to drought stress (Iuchi et al., 2001; Han et al., 2013a) and salt stress (Kim et al., 2013; Park et al., 2016). In addition, increasing evidence suggests that the length of the light period is important for the outcome of plant-pathogen interactions (e.g., Griebel and Zeier, 2008). Thus, photoperiod sensing enables plants to improve their responses to diverse environmental stresses (Figure 1). However, sudden changes in the photoperiod can also result in stress. Although the molecular mechanisms underlying this new abiotic stress form are not yet completely resolved (Nitschke et al., 2016, 2017; Abuelsoud et al., 2020; Frank et al., 2020), experiments revealed that changes in the photoperiod elicit stress reactions in *Arabidopsis* plants, which resemble responses to pathogen attack (Cortleven et al., 2021). The establishment of systemic acquired resistance (SAR) in plants forms an important defense against future pathogen attacks (Conrath et al., 2006). As photoperiod stress provokes similar effects, this might open new horizons for the use of altered photoperiods as a sustainable tool to alleviate pathogen infections and thereby decrease yield losses in horticulture. In the following chapters, we will address the above-mentioned topics in more detail.

## Molecular Mechanisms Involved in the Perception of Light and the Photoperiod

The perception of and response to photoperiods in plants require a sensing mechanism, which involves the detection of light (via photoreceptors or chloroplasts) and the measurement of time (via the circadian clock) (Jackson, 2009; Serrano-Bueno et al., 2021).

Light perception by photoreceptors and chloroplasts provides plants with comprehensive information concerning their surrounding light environment, such as quality (spectral composition, direction), quantity, intensity, and duration of incoming irradiation (Figure 1). In *Arabidopsis thaliana*, five photoreceptor families sense the light from different parts of the solar light spectrum: red/far-red light is detected by phytochromes (phyA to phyE). Blue light is perceived by cryptochromes (CRY1, CRY2, CRY3), phototropins (PHOT1, PHOT2), and F-box containing flavin-binding proteins ZEITLUPE (ZTL) and FLAVIN-BINDING KELCH REPEAT F-BOX1 (FKF1)/LOV KELCH PROTEIN2 (LKP2). UV light is sensed by the UVR8 photoreceptor (for review, see Sanchez et al., 2020; Roeber et al., 2021). All of the above-mentioned photoreceptor families are involved in the light entrainment of the circadian clock.

Besides the photoreceptors, chloroplasts operate as plant light sensors and respond to different photoperiods by altering their ultrastructure (Lepisto and Rintamaki, 2012). Chloroplasts of plants grown under long days exhibit smaller grana stacks and increased chlorophyll content. These features correspond to structural and photosynthetic characteristics typical of sun plants (Walters and Horton, 1995). Redox signals arising from chloroplasts determine the light intensity-dependent acclimation of plants (Pfannschmidt et al., 2009). Which signaling mechanisms are involved in the photoperiodic-dependent development of chloroplasts remains to be resolved. The redox state of the photosynthetic electron transport chain, ROS metabolism, and chloroplast-to-nucleus retrograde signaling are only few examples of possible pathways involved, all acting independent of the photoreceptors (for review, see Lepisto and Rintamaki, 2012; Feng et al., 2016). Besides the chloroplast ultrastructure, the photoperiod regulates the photosynthate partitioning to starch and the amount of carbohydrate (C) stored in chloroplasts (Zeeman et al., 2007). Under conditions where less C is available such as short photoperiods, a larger proportion of the fixed C is allocated into starch (Smith and Stitt, 2007). During the night the near-linear starch degradation is slowed down as compared to long-day-grown plants. This results in an almost but not completely exhausted starch content at dawn preventing C-starvation or C-excess at the end of the night period (Stitt and Zeeman, 2012; Moraes et al., 2019). This pattern of C-mobilization is robust across different photoperiods (Stitt and Zeeman, 2012; Moraes et al., 2019). Also here, the exact molecular mechanisms controlling the formation of starch under various photoperiods are not known, but possible feedback inhibition from the carbohydrate metabolism, redox regulation, transcriptional control of chloroplast enzymes, and circadian regulation might play a role.

**FIGURE 2:**
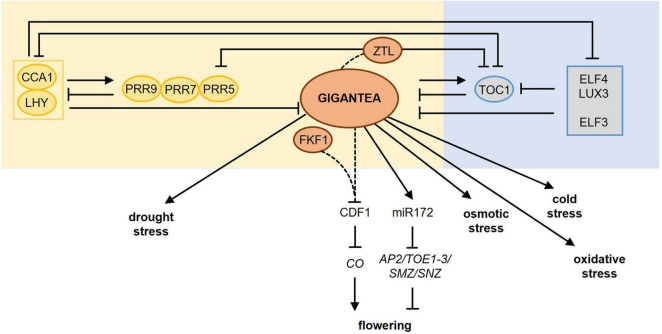
GIGANTEA plays a central role in photoperiod sensing and mediates the impact of photoperiod on stress responses. The circadian clock is an internal time-keeping mechanism involved in photoperiod sensing. The main regulatory components of the circadian clock are shown including their mutual transcription-translation feedback loops. *GIGANTEA* is expressed late in the afternoon and the protein improves the stability of ZEITLUPE (ZTL) upon blue light perception thereby targeting TOC1 and PRR5 for proteasomal degradation, reinforcing the entrainment of the clock. Upon blue light perception, GI interacts also with FKF1 causing the degradation of CYCLING DOF FACTOR1 (CDF1), which is a transcriptional repressor of *CONSTANS* (*CO*), encoding a central protein in photoperiod-dependent flowering. In addition, GI regulates the miR172-mediated post-transcriptional downregulation of several floral repressor genes. Besides its role in photoperiod-dependent flowering, GI has a central role in the photoperiod-dependent plant responses to drought, osmotic, cold, and oxidative stress. Dashed lines mark protein–protein interactions upon blue light perception. For more detailed information about the different pathways, please refer to section “Molecular Mechanisms Involved in the Perception of Light and the Photoperiod.” AP2, APETALA2; CCA1, CIRCADIAN CLOCK ASSOCIATED1; LHY, LATE ELONGATED HYPOCOTYL; PRR, PSEUDO-RESPONSE REGULATOR, TOC1, TIMING OF CAB EXPRESSION1; ELF3, EARLY FLOWERING3; ELF4, EARLY FLOWERING4; LUX, LUX ARRHYTHMO; FKF1, FLAVIN-BINDING KELCH REPEAT F-BOX1; TOE1, TARGET OF EAT1; TOE2, TARGET OF EAT2; SMZ, SCHLAFMÜTZE; SNZ, SCHNARCHZAPFEN.

The circadian clock enables plants to measure time by an endogenous time-keeping mechanism (Hsu and Harmer, 2014; Figure 2). The clock is set through daily entrainment, especially by light and temperature, in order to adjust the internal rhythm (McClung, 2006). In *Arabidopsis thaliana*, the circadian clock consists of multiple interlocked transcription-translation feedback loops (Hsu and Harmer, 2014). The MYB-domain transcription factor genes *CIRCADIAN CLOCK ASSOCIATED1* (*CCA1*) and *LATE ELONGATED HYPOCOTYL* (*LHY*) are expressed in the morning (Schaffer et al., 1998; Wang and Tobin, 1998) and repress the expression of *TIMING OF CAB EXPRESSION1* (*TOC1*) during the day (Alabadi et al., 2001). In turn, TOC1 represses the transcription of *CCA1* and *LHY* (Gendron et al., 2012). Late at night, *TOC1* transcription is down-regulated by an Evening Complex (EC), which is composed of three proteins: EARLY FLOWERING3 (ELF3), ELF4, and LUX ARRHYTHMO (LUX). This down-regulation enables transcription of *LHY* and *CCA1* to resume the following dawn. *PSEUDO-RESPONSE REGULATOR9* (*PRR9*), *PRR7*, and *PRR5* are expressed in consecutive waves throughout the day and repress *CCA1* and *LHY* expression (Nakamichi et al., 2012). Additional rhythmically expressed transcriptional activators, such as REVEILLE4 (RVE4), RVE6, and RVE8, the LIGHT-REGULATED WD1 (LWD1) and LWD2 proteins, and the transcription factors NIGHT LIGHT-INDUCIBLE AND CLOCK-REGULATED GENE1 (LNK1) and LNK2 also contribute to the clock function (Rawat et al., 2011; Rugnone et al., 2013). The circadian clock contributes to the plants’ ability to respond to various environmental stresses but there is also a reciprocal influence of abiotic stresses on the clock function. More information about this can be found in the reviews of Sanchez et al. (2011), Kiełbowicz-Matuk and Czarnecka (2014), Grundy et al. (2015), Seo and Mas (2015), and Sharma et al. (2021). A novel webtool to investigate the transcriptional networks regulated by light and the circadian clock has been launched recently (de los Reyes et al., 2020). With ATTRACTOR (*Arabidopsis Thaliana* TRanscriptionAl Circadian neTwORk^1^), target genes of circadian regulators can be identified. This might contribute to a better understanding of the interaction between the circadian clock and plant responses to environmental stresses.

One of the circadian clock-controlled genes that have a crucial role in the photoperiod sensing mechanism is GIGANTEA (GI) (Fowler et al., 1999). It encodes a large single-gene encoded protein with a chaperone activity (Cha et al., 2017). Upon blue light perception, the stability of the F-box protein ZEITLUPE (ZTL) improves due to interaction with GI. ZTL is an evening-phased E3 ubiquitin ligase targeting the clock components TOC1 and PRR5 for proteasomal degradation (Mas et al., 2003; Kiba et al., 2007). GI protein abundance peaks in the evening, thereby maintaining ZTL abundance high. Consequently, high amplitude oscillations of TOC1 and PRR5 are sustained (Kim et al., 2007). This reinforces the entrainment of the clock resulting in the correct setting of the phase of clock output genes such as *CONSTANS (CO)*, encoding a central protein in photoperiod-dependent flowering (Suarez-Lopez et al., 2001; Shim et al., 2017). GI also interacts with FKF1 upon blue light perception causing the degradation of CYCLING DOF FACTOR1 (CDF1), which is a transcriptional repressor of *CO* (Figure 2; Sawa et al., 2007). The synchronization of the correct timing of protein stabilization during long days with the circadian-regulated expression of *FKF1*, *GI*, and *CDF1* is essential for photoperiodic responses, such as flowering. Interesting to mention is that the CO-FT-GI-CDF hub is conserved among distantly related flowering plants (Serrano-Bueno et al., 2021).

GI also regulates the maturation of miR172 (Jung et al., 2007), which targets *APETALA2* (*AP2*) and the *AP2*-like genes *TARGET OF EAT1* (*TOE1*), *TOE2*, *TOE3*, *SCHLAFMÜTZE* (*SM*Z), and *SCHNARCHZAPFEN* (*SNZ*) (Figure 2). The miR172-mediated posttranscriptional downregulation of these floral repressors regulates flowering time and floral development in the shoot apical meristem (Mathieu et al., 2009) depending on the age of the plants (Aukerman and Sakai, 2003). In addition, GI controls the circadian clock-mediated photoperiod sensing together with EARLY FLOWERING3 (ELF3). In their absence, the circadian clock fails to properly respond to light signals, resulting in the breakdown of the photoperiod sensing mechanism (Anwer et al., 2020).

GI plays not only a central role in the photoperiod sensing mechanism but is also involved in mediating the impact of the photoperiod in response to diverse stresses (Figure 2), e.g., drought, oxidative, osmotic, and cold stress (Cao et al., 2005; Fornara et al., 2015), as will be outlined further below.

## The Photoperiod Influences Responses to Abiotic and Biotic Stresses

### Photoperiod and Freezing Tolerance

**FIGURE 3:**
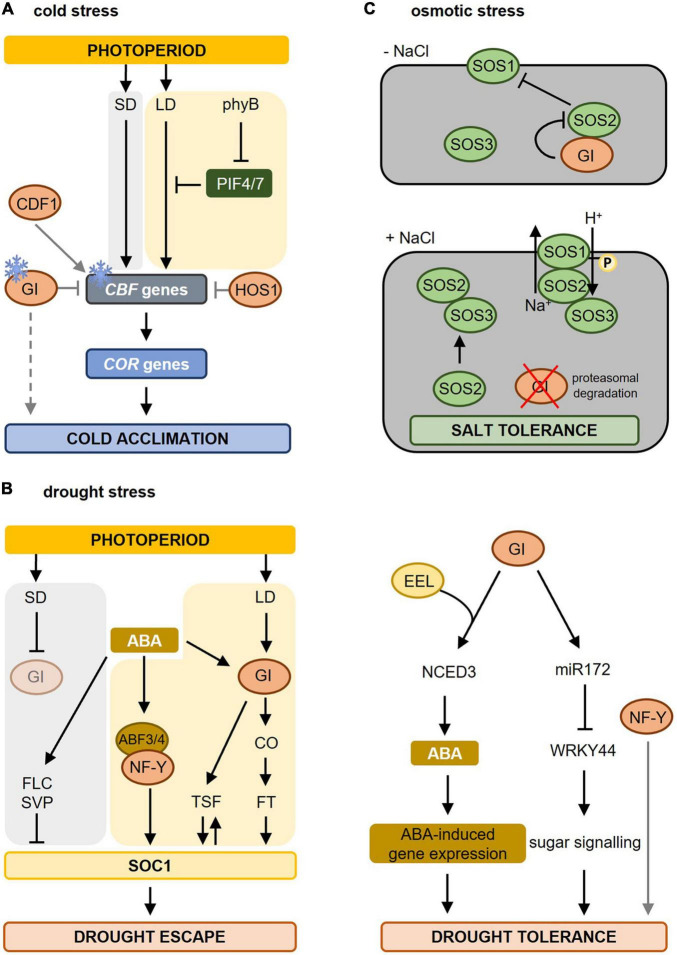
Molecular mechanisms involved in photoperiod-dependent responses to cold, drought, and osmotic stress. **(A)** During cold stress (indicated by the ice crystal), *CBF* gene expression is upregulated and induces the expression of *COR* genes resulting in cold acclimation. Under short-day (SD) conditions, *CBF* genes are strongly induced causing cold acclimation. Under warmer long-day (LD) conditions, *PIF4* and *PIF7*, which are under the control of phyB, are higher expressed resulting in an inhibition of *CBF* gene expression. As days shorten, e.g., during autumn, this repression falls away resulting in cold acclimation. GI is also induced by colder temperatures and blocks the *CBF* genes, whereas CDF1 promotes the expression of *CBF*. GI also promotes freezing tolerance in a CBF-independent manner (dashed line). In addition, HOS1, another photoperiod-dependent flowering-inducing component inhibits *CBF* gene expression thereby blocking cold acclimation. Figure adapted from Roeber et al. (2021). **(B)** Drought stress results in biosynthesis of abscisic acid (ABA) leading to ABA-dependent gene regulation causing drought escape (left) and drought tolerance (right). The increased ABA levels promote earlier flowering (drought escape, left part) under LD but not under SD conditions. Under LD conditions, GI is activated and activates the expression of florigen genes (*TSF* and *FT* via CO) triggering the activation of *SOC1* and inducing flowering. SOC1 in turn contributes to *TSF* upregulation boosting SOC1 activity. ABA also induces the expression and activity of ABF3 and ABF4. ABF3/4, together with their interacting partner, the NF-Y complex, binds to the *SOC1* promoter and promotes its expression to accelerate flowering during drought escape. Under SD conditions, delay of flowering occurs during drought stress due to enhanced activity of repressors like FLC and SVP on *SOC1* transcription. Under these SD conditions, GI is not activated (pale circle). Adapted from Riboni et al. (2013, 2016) and Hwang et al. (2019). In the drought tolerance signaling pathway (right part), GI forms a complex with EEL (ENHANCED EM LEVEL) thereby upregulating the diurnal expression of *NCED3* (*NINE-CIS-EPOXYCAROTENOID DIOXYGENASE3*) encoding a rate-limiting enzyme in ABA synthesis. Furthermore, interaction between GI and miR172 results in a reduction of *WRKY44* expression promoting sugar signaling and drought tolerance. Besides GI, also NUCLEAR FACTOR-Y (NF-Y) promotes drought tolerance. **(C)** In the absence of salt stress (-NaCl) GI represses SOS2 thus blocking the SOS pathway. Upon salt stress (+ NaCl), the proteasomal degradation of GI is promoted, releasing SOS2. Free SOS2 interacts with SOS3 to form an active SOS2–SOS3 protein kinase complex that translocates to the plasma membrane causing the phosphorylation and activation of SOS1 resulting in salt stress tolerance. Adapted from Kim et al. (2013). For more information concerning the different pathways, please refer to section “Photoperiod and Freezing Tolerance” for cold stress, section “Photoperiod and Drought Stress” for drought stress, and section “Photoperiod and Osmotic Stress” for osmotic stress. Yellow background marks pathways taking place in LD conditions, a gray background indicates pathways during SD conditions. Gray lines mark the direct influence of specific photoperiod sensing components on stress responses. LD, long day; SD, short day; phyB, phytochrome B; PIF, PHYTOCHROME INTERACTING FACTOR; CBF, C-repeat/dehydration-responsive element-binding factor; COR, COLD-REGULATED; GI, GIGANTEA; HOS1, HIGH EXPRESSION OF OSMOTICALLY RESPONSIVE GENE1; CDF1, CYCLING DOF FACTOR1; ABA, abscisic acid; ABF, abscisic acid binding factor; CO, CONSTANS; TSF, TWIN SISTER OF FT; SOC1, SUPPRESSOR OF OVEREXPRESSOR OF CONSTANS; FLC, FLOWERING LOCUS C; SVP, SHORT VEGETATIVE PHASE; SOS, SALT OVERLAY SENSITIVE.

One of the best-known stress tolerances depending on the photoperiod is freezing tolerance (Figure 3A). The shortening of day length sensed by plants in autumn anticipates the effect of colder temperatures in winter and causes an increased freezing tolerance (Lee and Thomashow, 2012). For example, red-osier dogwood (*Cornus sericea*) responds to a shortening of the photoperiod by a decrease of the stem water content, which results in an increased freezing tolerance (Karlson et al., 2003). In hybrid aspen, the phyA-mediated apical bud formation under short days is the main switch turning metabolism from vegetative growth to dormancy and inducing freezing tolerance (Welling et al., 2002).

Increased freezing tolerance caused by shortening of the photoperiod also occurs in *Arabidopsis thaliana*. Geographical distant accessions of *Arabidopsis* exhibit differences in freezing tolerance, which can be related to the photoperiod conditions they are geographically exposed to (Alonso-Blanco et al., 2005). The C-repeat/dehydration-responsive element-binding factor (CBF/DREB) signaling cascade is the central molecular mechanism mediating these differences in response to day length. Cold temperatures stimulate the *CBF* genes resulting in the induction of COLD-REGULATED (*COR*) genes leading to freezing tolerance (Thomashow, 2010; Pareek et al., 2017). Under long days, the *CBF* regulon is repressed by phyB, PHYTOCHROME INTERACTING FACTOR4 (PIF4), and PIF7, which causes less freezing tolerance. Shortening of the days during autumn relieves this repression causing an increased expression of the *CBF* genes, thereby preparing plants for upcoming colder temperatures (Figure 3A; Lee and Thomashow, 2012).

Among the components involved in photoperiodic flowering, the GI-CDF module regulates also freezing tolerance in *Arabidopsis* (Fornara et al., 2015). *GI* expression is induced by cold (Fowler and Thomashow, 2002; Cao et al., 2005) and many cold-regulated genes in *Arabidopsis* are co-regulated by GI and CDFs (Figure 3A). In *gi-100* mutants, mRNA of *COR* genes was present at higher levels than in wild type correlating with enhanced expression of *CDF1, CDF2, CDF3 and CDF5* and increased freezing and oxidative stress tolerance. Consequently, this increase in *COR* gene expression was suppressed in *gi-100 cdf1235* mutants (Fornara et al., 2015). In contrast, Cao et al. (2005) found that *gi-3* mutants are hypersensitive to freezing. As no differences were found in the transcript levels of *CBF* genes upon cold stress, it was concluded that GI acts in a CBF-independent manner to promote freezing tolerance by altering the carbohydrate metabolism. The exact mechanisms are still unclear (Cao et al., 2005, 2006). Such divergences may be due to the use of *gi* mutant alleles in different ecotypes and/or different assay conditions (Fornara et al., 2015). However, *gi* loss-of-function mutants of *Brassica rapa* plants show increased freezing tolerance suggesting that the role of GI in resistance to freezing stress is conserved between species (Xie et al., 2015).

Another component involved in regulating both photoperiod flowering and freezing tolerance is HOS1 (HIGH EXPRESSION OF OSMOTICALLY RESPONSIVE GENE1) (Figure 3A). HOS1 encodes a RING finger-containing E3 ubiquitin ligase controlling the abundance of CO thereby ensuring that the CO-dependent activation of *FT* occurs only when the light period reaches a certain length (Lazaro et al., 2012). HOS1 negatively regulates cold acclimation by mediating the ubiquitination and proteasomal degradation of ICE1 (INDUCER OF CBF EXPRESSION1) and thus negatively regulates the CBF regulon (Ishitani et al., 1998; Dong et al., 2006; Lee and Thomashow, 2012).

### Photoperiod and Drought Stress

Drought has detrimental effects on plants limiting their performance and productivity. Upon the perception of drought signals, the endogenous abscisic acid (ABA) level increases resulting in closure of the stomata in order to decrease water loss via transpiration (Outlaw, 2003).

The drought escape is an adaptive strategy of plants to accelerate reproductive development (i.e., flowering) under drought stress (Figure 3B). This allows plants to finish their life cycle before severe stress results in lethality (McKay et al., 2003). Drought escape only occurs under inductive long-day conditions involving the photoperiodic response gene *GI* and the florigen genes *FLOWERING LOCUS T* (*FT*) and *TWIN SISTER OF FT* (*TSF*) (Riboni et al., 2013). Drought stress releases the transcriptional repression at the *FT/TSF* promotors in an ABA- and photoperiod (via GI)-dependent manner thereby promoting transcriptional upregulation of the florigen genes (Riboni et al., 2013). The ABA-dependent activation of *FT*, but not of *TSF*, requires CO (Riboni et al., 2016). Increased florigen levels trigger the activation of the floral integrator SUPPRESSOR OF OVEREXPRESSOR OF CONSTANS (SOC1) thereby initiating flowering. SOC1 activation contributes to *TSF* upregulation thus further increasing the florigen levels (Riboni et al., 2013). Under short-day conditions, ABA delays flowering under drought stress due to the repressive action of SHORT VEGETATIVE PHASE (SVP)/FLOWERING LOCUS C (FLC) on *SOC1* (Riboni et al., 2016). Also the NUCLEAR FACTOR Y (NF-Y) subunit c, belonging to a family of transcription factors known to be involved in photoperiod-dependent flowering (Kumimoto et al., 2008, 2010), is implicated in drought escape. The ABA-response element (ABRE)-binding factors (ABFs) interact with NF-Y subunit c-3/4/9, thereby inducing *SOC1* to promote flowering (Hwang et al., 2019). Besides *Arabidopsis*, also wheat and barley have a drought escape strategy (McMaster and Wilhelm, 2003; Gol et al., 2021), just like *Avena barbata* (Sherrard and Maherali, 2006) and *Brassica rapus* (Franks, 2011). Other species, such as rice, delay flowering upon drought stress to resume its life cycle when the stress is over (Galbiati et al., 2016). Also here, primary integrators of day length provide a molecular connection between stress and the photoperiodic flowering pathway. Taken together, drought escape is a photoperiod-depend developmental response as it is the direct consequence of the perception of the long-day photoperiod during drought stress.

Besides their role during drought escape, the photoperiod sensing components GI and NF-Y are known to additionally influence drought tolerance without any direct link to the perception of the photoperiod (Figure 3B). The synthesis and signaling of ABA are at least partially under photoperiodic control (Zeevaart, 1971). A recent study (Baek et al., 2020) revealed that GI forms a complex with the bZIP transcription factor ENHANCED EM LEVEL (EEL) involved in ABA signaling responses to regulate the expression of *NINE-CIS-EPOXYCAROTENOID DIOXYGENASE3* (*NCED3*). *NCED3* encodes a rate-limiting enzyme in ABA synthesis (Iuchi et al., 2001). The GI-EEL complex positively regulates the diurnal ABA synthesis by binding to the ABA-responsive element motif in the *NCED3* gene promotor resulting in increased ABA synthesis and improved drought tolerance (Baek et al., 2020). The abundance of *NCED3* transcript and ABA content decreased in *gi-1* and *eel* mutants under dehydration, which correlates with their dehydration-sensitive phenotype (Baek et al., 2020). These results indicate that GI and EEL together enhance the plant tolerance to drought by regulating ABA homeostasis.

Another study described a role for GI during the drought stress response (Figure 3B; Han et al., 2013a). Upon drought stress, both level and function of mature miR172 are upregulated, with *miRNA172e* showing the strongest response to drought stress (Han et al., 2013a). Under long days and drought conditions, GI promotes the processing of pre-miRNA172 resulting in a suppression of *WRKY44*, which leads to drought tolerance. The exact underlying mechanism is not fully understood but might relate to sugar metabolism (Han et al., 2013a).

NF-Y transcription factors (Figure 3B) have been shown to improve drought tolerance in *Arabidopsis* (Li et al., 2013; Ni et al., 2013), maize (Nelson et al., 2007; Su et al., 2018), poplar (Han et al., 2013b), rice (Chen et al., 2015), and citrus (Pereira et al., 2018). In *Arabidopsis*, overexpression of *NF-YA5* improved drought tolerance and micro-array analysis revealed that oxidative stress-responsive genes are strongly upregulated upon drought stress (Li et al., 2008). Transgenic *Arabidopsis* plants overexpressing the soybean *NF-YA3* gene exhibited an increased expression of ABA biosynthesis, signaling, and stress-responsive genes (Ni et al., 2013). While this study suggested an ABA-dependent signaling resulting in improved drought resistance, overexpression of *NF-YB1* in *Arabidopsis* also enhanced plant drought resistance independent of ABA signaling (Nelson et al., 2007). Besides their role in drought tolerance, overexpression of *NF-Y* transcription factors genes also improves freezing tolerance (Shi and Chan, 2014) and salt stress resistance (Li et al., 2008).

### Photoperiod and Osmotic Stress

Osmotic stress leads to desiccation, due to the high osmotic potential of saline soils, and inhibits plant growth and development (Munns, 2002; Mahajan and Tuteja, 2005). To cope with salinity or osmotic stress, plants have developed adaptation strategies, such as decreasing the water loss by stomata closure, decreasing their growth, and activating antioxidant systems (Munns and Tester, 2008). The salt overly sensitive (SOS) pathway forms the first line of defense to salt stress in *Arabidopsis* plants (Ji et al., 2013). The SOS pathway, which depends on SOS1, SOS2, and SOS3, has been shown to regulate cellular signaling during salt stress to achieve ion homeostasis. *SOS1* encodes a Na^+^/H^+^-antiporter located at the plant cell plasma membrane, which is responsible for the efflux of Na^+^ from the cytoplasm to the apoplast. SOS1 is activated by the calcium-regulated SOS2-SOS3 protein kinase complex (Shi et al., 2000; Qiu et al., 2002).

GI has been shown to be a major component of the salt stress adaptation pathway (Figure 3C; Kim et al., 2013). *gi* mutants are salt stress tolerant, while *GI* overexpression lines are extremely salt-sensitive. The underlying mechanism was revealed by Kim et al. (2013). Under non-stress conditions, GI prevents SOS2 from activating SOS1, thereby retaining the SOS system in a resting state. Upon salt stress, GI is degraded releasing SOS2 for interaction with SOS3, which causes in turn the activation of SOS1 to re-establish ion homeostasis (Kim et al., 2013; Park et al., 2016). No direct effect of the photoperiod on plant performance under salt stress is known. The involvement of the photoperiod-sensitive GI in the SOS pathway, however, suggests that the photoperiod might have a strong impact on salt stress tolerance.

### Photoperiod and Biotic Stress Responses

Increasing evidence indicates that the length of the light period is also important for the response to diverse biotic stresses, including the responses to viruses, bacteria, and fungi (Table 1). The first observations showing that the photoperiod influences the response to pathogen infection were from Cecchini et al. (2002). They found that short-day-grown *Arabidopsis* (L*er*) plants developed stronger disease symptoms than long-day-grown plants after infection with cauliflower mosaic virus (CaMV Cabb B-JI), although the virus replication was even higher under long-day conditions. Later on, Griebel and Zeier (2008) demonstrated that for *Arabidopsis* plants grown in different but constant light-dark cycles, the early disease resistance of *Arabidopsis* plants inoculated with *Pseudomonas syringae* pv. *maculicola* (*Psm*) ES4326 harboring the *avrRpm1* avirulence gene is positively correlated with the length of the light period, underpinning the importance of the photoperiod. They also showed that the concentration of salicylic acid (SA) accumulated in *Psm avrRpm1-*infected *Arabidopsis* leaves, the early expression of the SA-regulated defense gene *PATHOGENESIS-RELATED GENE1* (*PR1*), and the magnitude of the hypersensitive response-induced lesion formation are influenced by the duration of the light period. Long-day-entrained *Arabidopsis* plants exposed to constant light were less susceptible to infections with virulent *Hyaloperonospora parasitica* isolate Noco2 (Evrard et al., 2009) or *P. syringae* pv. *tomato* DC3000 (Cortleven et al., 2021). Transferring *Arabidopsis* from a short to a long photoperiod enhanced the resistance to the necrotrophic fungus *Botrytis cinerea* (Cagnola et al., 2018) and the hemibiotrophic fungus *Pyricularia oryzae* (syn. *Magnaporthe oryzae*) (Shimizu et al., 2021). In the latter case, the outcome of early plant–pathogen interactions was influenced by the length of the photoperiod following inoculation. The plant resistance to fungus penetration was enhanced, if a light period followed evening inoculations instead of the normal dark period (Shimizu et al., 2021).

**TABLE 1:** The photoperiod affects in plants the response to biotic stress.

Plant species	Light conditions	Plant pathogen	Effects under longer photoperiods	References
*A. thaliana* L*er*	SD-entrained plants (10 h L/14 h D) or LD-entrained plants (16 h L/8 h D)	Cauliflower mosaic virus	Lower susceptibility to infection when plants were LD-entrained	Cecchini et al., 2002
*Rhododendron* cv. Elizabeth	Cut leaves were infected and incubated under SD (8 h L/16 h D) or LD (16 h L/8 h D)	*Erysiphe* sp.	Lower susceptibility to infection when leaves were incubated under LD	Kenyon et al., 2002
*A. thaliana* Col-0	LD-entrained plants (14 h L/10 h D) transferred to constant light (3 days)	*Hyaloperono-spora parasitica* Noco2	Lower susceptibility to infection when pre-treated with constant light; responsiveness of the promoter motif FORC^A^ to defense stimuli is regulated by duration of light period	Evrard et al., 2009
*A. thaliana* Col-0	SD-entrained plants (9 h L/15 h D) infected at different times of the day, thereby influencing the light availability after infection	*Pseudomonas syringae* pv. *maculicola* harboring the *avrRpm1* avirulence gene	Lower susceptibility to infection in the morning; length of light period during early plant-pathogen interaction determines salicylic acid production, *PR1* accumulation and formation of hypersensitive response	Griebel and Zeier, 2008
*A. thaliana* Col-0	SD-entrained plants (8 h L/16 h D) transferred to LDs (16 h L/8 h D)	*Botrytis cinerea*	Lower susceptibility to infection when transferred to LDs; jasmonic acid-related plant defense responses are enhanced under LDs	Cagnola et al., 2018
*Solanum lycopersicum* cv. Ailsa Craig	Plants entrained in a 12 h L/12 h D photoperiod treated with nightly red light replacing the normal dark period and thereby extending the total light period	*Pseudomonas syringae* pv. *tomato* DC3000	Lower susceptibility to infection; the enhanced plant defense correlated with the accumulation of salicylic acid, the transcriptional induction of defense-related genes and alleviation of pathogen-induced cell death	Yang et al., 2015
*Zea mays Hm1* ^A^	Plants entrained in a 12 h L/12 h D photoperiod were infected and subsequently exposed to the 12 h L/12 h D photoperiod or to LDs (18 h L/6 h D)	*Cochliobolus carbonum* race 1 (CCR1)	Lower susceptibility to infection when transferred to LDs (compared to plants kept in 12 h L/12 h D photoperiods)	Marla et al., 2018
*Fragaria ananassa* cv. Elsanta	Plant leaf discs were infected and incubated in presence or absence of light	*Botrytis cinerea*	Lower susceptibility to infection when leaf discs were incubated in light; red light incubation further decreased the susceptibility	Meng et al., 2020
*A. thaliana* Col-0	SD-entrained plants (8 h L/16 h D) transferred 24 h or 8 h prolonged light period	*Pseudomonas syringae* pv. *tomato* DC3000	Lower susceptibility to infection when pre-treated with prolonged light periods	Cortleven et al., 2021
*Brassica juncea*	Entrainment of plants in four different photoperiods (SD with 8 h L/16 h D; 12 h L/12 h D; LD with 16 h L/8 h D; constant light)	*Alternaria brassicicola*	Lower susceptibility to infection when grown in 12 h L/12 h D or in constant light photoperiods; largest necrosis after infection were observed in LD-entrained plants	Macioszek et al., 2021
*A. thaliana* Col-0	SD-entrained plants (9 h L/15 h D) infected at different times of the day; subsequent transfer to constant light or darkness	*Pyricularia oryzae* syn. *Magnaporthe oryzae*	Lower susceptibility to infection when the infection was followed by a light period	Shimizu et al., 2021

The length of the light period influences plant responses to biotic stresses on the transcriptional level. Evrard et al. (2009) demonstrated that in *Arabidopsis* plants grown under 14 h day/10 h night cycles or under the same conditions but followed by 3 days of darkness, the transcriptional activity mediated by the hexameric promoter motif FORC^A^ is suppressed by defense-related stimuli. In contrast, in constant light, the FORC^A^-mediated gene expression is enhanced resulting in increased defense. More generally, Baerenfaller et al. (2015) showed that the abundance of transcripts for biotic stress responses increased in *Arabidopsis* grown under long photoperiods compared to plants cultivated under short-day conditions. Similarly, Cagnola et al. (2018) revealed by transcriptome analysis of *A. thaliana* transferred from short- to long-day conditions that long photoperiods enhance the jasmonic acid (JA)-related plant defense responses.

An improved resistance to biotic stimuli under long photoperiods is also observed in other plant species than *Arabidopsis*. Kenyon et al. (2002) reported that long photoperiods enhanced the resistance of *Rhododendron* cv. Elizabeth cut leaves, as fewer hyphae of the fungus *Erysiphe* sp. were produced than under short photoperiods. In tomato plants (*Solanum lycopersicum* cv. Ailsa Craig), nightly red light treatment (replacing the normal dark period and thereby extending the duration of the total light period) enhanced the plant resistance against *P. syringae* pv. *tomato* DC3000 infection. The increased resistance was correlated with the accumulation of SA, increased abundance of defense-related transcripts and alleviated pathogen-induced cell death (Yang et al., 2015). The resistance of maize *Hm1*^A^ seedlings (containing a partial loss-of-function mutation in the *Hm1* gene, encoding HC-toxin reductase inactivating the HC-toxin produced by *Cochliobolus carbonum*, which causes leaf spot in maize) inoculated with *C. carbonum* race 1 was enhanced in plants grown after infection in extended light periods and might correlate to the energy status of the plant (Marla et al., 2018). Similarly, strawberry (*Fragaria ananassa* cv. Elsanta) plants inoculated with *B. cinerea* developed stronger disease symptoms, if plants were transferred to darkness after infection compared to plants kept under their normal light conditions (Meng et al., 2020). Macioszek et al. (2021) observed that growth under short-day conditions results in increased necrosis formation in *Brassica juncea* plants infected with the necrotrophic fungus *Alternaria brassicicola*.

Overall, these publications clearly highlight the importance of the duration of the photoperiod in plant responses to diverse biotic stresses. Generally, a longer photoperiod causes increased biotic stress resistance. Whether this is causally linked to an improved energy state of the plants under longer photoperiods has not been discussed in the above-mentioned publications, but would be an interesting direction for future research. The role of another parameter influenced by the length of the photoperiod, the plant redox state, will be discussed in the following chapter.

## Photoperiod and the Plant Redox State

A possible reason for the impact of the photoperiod on responses to diverse stresses is its regulatory influence on the plant redox state (for review see Shim and Imaizumi, 2015). The plant redox state and regulation of redox reactions are connected with levels of reactive oxygen species (ROS). ROS have previously been considered only as toxic by-products of aerobic metabolism, but recent research highlights their importance as plant signaling molecules (for review see Mittler, 2017). In addition, ROS cause post-translational modifications of cysteine residues in redox-sensitive proteins and thus interfere with the redox regulatory network of the cell allowing fast responses of plants to environmental cues (Cejudo et al., 2021).

ROS include several oxygen-containing molecules, such as singlet oxygen (^1^O_2_), superoxide (O_2_^–^), hydroxyl radicals (OH^–^), and hydrogen peroxide (H_2_O_2_) (Shim and Imaizumi, 2015; Mittler, 2017). Of these, especially H_2_O_2_ levels are regulated by the photoperiod. In the algae *Chlamydomonas reinhardtii*, elevated H_2_O_2_ levels in the presence of light result from slower H_2_O_2_ degradation due to light-dependent inactivation of catalases (Shao et al., 2008). Similar observations were made in rye (*Secale cereale*) leaves (Hertwig et al., 1992).

Experiments with *Arabidopsis catalase2* (*cat2*) mutants exposed to different day lengths were particularly informative about the influence of the photoperiod on oxidative stress responses. The photoperiod, in which the *cat2* mutants are grown, is decisive for the oxidative stress response and regulates H_2_O_2_-induced gene expression as well as the severity of the cell death phenotype (Queval et al., 2007; Chaouch et al., 2010; Shim and Imaizumi, 2015). While short-day-grown *cat2* mutants do not display any lesions, lesion formation is visible in long day-grown *cat2* mutants pointing to elevated H_2_O_2_ levels. Further analysis revealed that increased peroxisomal H_2_O_2_ in *cat2* triggers pathogen defense responses and enhances the plants’ resistance in a photoperiod-dependent manner. Also *lesion simulating disease1* (*lsd1*) mutants formed lesions only in long photoperiods (Mateo et al., 2004; Shim and Imaizumi, 2015). LSD1 is a catalase-interacting protein, regulating catalase activity, as a consequence, catalase activity is decreased in *lsd1* mutants. *LSD1* and *CATALASE* genes interact genetically and their encoded proteins are part of a protein complex, which plays an important role in regulating programmed cell death (PCD) (Li et al., 2013).

Similar as observed for catalase, also other enzymes involved in ROS detoxification including ascorbate peroxidase and NAD-malate dehydrogenase showed in *Arabidopsis* a higher activity under long photoperiods (Becker et al., 2006). GI was shown to be a negative regulator of the expression of genes encoding enzymes detoxifying ROS. Indeed, the increased tolerance of *gi-3* mutants to oxidative stress is caused by the constitutive activation of *SUPEROXIDE DISMUTASE* (*SOD*) and *ASCORBATE PEROXIDASE* (*APX*) genes (Cao et al., 2006).

Together these publications support a photoperiod-dependent regulation of the plant redox state. Photoperiod sensing is linked to redox regulation, allowing efficient light usage and redox balancing in short days and preventing oxidative damage in long days (Becker et al., 2006). Especially catalases but also SOD and APX seem to be important factors exerting photoperiod information on redox regulation (Shim and Imaizumi, 2015).

## Changes in the Photoperiod Cause Stress

**FIGURE 4:**
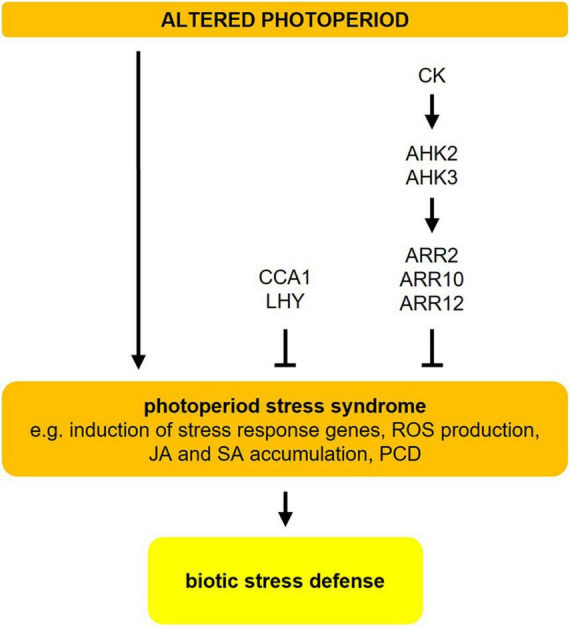
Photoperiod stress in *Arabidopsis*. Changes in the photoperiod, i.e., prolongation of the light period, result in a photoperiod stress syndrome, which is characterized by induction of stress response genes, ROS production, accumulation of jasmonic acid (JA) and salicylic acid (SA), and eventually programmed cell death (PCD). Both, cytokinin (CK) (mostly *trans*-zeatin) and CCA1/LHY are negative regulators of photoperiod stress. Photoperiod stress elicits a transcriptional response that resembles the response to ozone stress and pathogen infection. The resistance to an infection with *P. syringae* pv. *tomato* DC3000 is increased after a preceding photoperiod stress event. For more detailed information about the different pathways, please refer to sections “Changes in the Photoperiod Cause Stress” and “Photoperiod Stress Elicits a Similar Response as Pathogen Infection.” The figure has been adapted from Roeber et al. (2021).

Sudden changes in the photoperiod, in particular its prolongation, cause photoperiod stress in short-day-adapted *A. thaliana* plants (Nitschke et al., 2016, 2017; Figure 4). The photoperiod stress response, which was originally observed after a prolongation of the light period by 24 h, is characterized by a typical course of events: During the night following an extended light period, the expression of stress marker genes, such as *ZINC FINGER of ARABIDOPSIS THALIANA12* (*ZAT12*) and *BON ASSOCIATED PROTEIN1* (*BAP1*), is induced, the concentration of the stress hormones JA and SA increase and oxidative stress occurs. The nightly increase in oxidative stress coincides with a strong decrease in the ascorbic acid (ASC) redox state and the formation of peroxides. The peroxide formation is associated with an increase of *PEROXIDASE* (*PRX*) gene expression as well as enhanced PRX and decreased catalase activities (Abuelsoud et al., 2020). During the next day, the photosystem II maximum quantum efficiency decreases, and eventually PCD ensues in the leaves (Nitschke et al., 2016, 2017).

Photoperiod stress was first noted in cytokinin (CK)-deficient *Arabidopsis* plants, which show a particularly strong stress response. Among the CKs, especially *trans*-zeatin has a protective function acting through the ARABIDOPSIS HISTIDINE KINASE3 (AHK3) receptor and the transcriptional regulators ARABIDOPSIS RESPONSE REGULATOR2 (ARR2), ARR10, and ARR12 (Figure 4; Frank et al., 2020). Certain clock mutants (e.g., *cca1 lhy*, *elf3*) also show a stronger molecular and phenotypical response to sudden prolongations of the photoperiod. Both photoperiod stress-sensitive clock mutants and CK-deficient plants have a lower expression or impaired function of CCA1 and LHY, two key regulators of the circadian clock. This indicates that a functional clock is required to cope with photoperiod stress (Figure 4; Nitschke et al., 2016, 2017).

In short-day-entrained *Arabidopsis* plants, a prolongation of the light period by only 4 h is sufficient to induce the production of ROS and the expression of stress marker genes during the following night. Longer prolongations of the light phase induce a gradually stronger stress response, which indicates that light duration has an impact on the strength of the photoperiod stress response (Abuelsoud et al., 2020). Shorter prolongations of the light period, which cause lower stress levels, are perceived as not harmful and may present a beneficial stress (eustress), while higher stress levels (by longer prolongations) induce a true stress (distress) (Krasensky-Wrzaczek and Kangasjarvi, 2018).

## Photoperiod Stress Elicits a Similar Response as Pathogen Infection

RNA-seq analysis of 5-weeks-old short-day grown *Arabidopsis* plants exposed to a 24 h-prolongation of the light period revealed that photoperiod stress causes massive time-dependent transcriptomic changes during the night following the prolonged light period (Cortleven et al., 2021). Among the photoperiod stress-responsive genes are numerous genes related to ROS. The photoperiod stress transcript profile resembles that caused by ozone stress and pathogen attacks, which commonly elicit an apoplastic oxidative burst. Moreover, both SA and camalexin levels increased and transcript levels of genes involved in SA biosynthesis, such as *ISOCHORISMATE SYNTHASE1* (*ICS1*), and genes involved in SAR, such as *PATHOGENESIS RELATED1* (*PR1*) were induced by photoperiod stress (Cortleven et al., 2021).

Interestingly, photoperiod stress pre-treated wild-type plants showed less *P. syringae* pv. *tomato* DC3000 colony-forming units after infection in comparison to non-photoperiod stress-treated plants. This indicates that not only similar molecular pathways are activated in response to photoperiod stress and pathogen attack, but that photoperiod stress pre-treatment leads to improved pathogen immunity in *Arabidopsis* plants without an actual pathogen attack (Cortleven et al., 2021). Other reports support this conclusion. In tomato, it was shown that nightly red light treatment improved the resistance against *P. syringae* pv. *tomato* DC3000 (Yang et al., 2015). The improved resistance was associated with increased SA levels and the induction of defense-related genes, which is typical of a photoperiod stress response (Cortleven et al., 2021). The transfer of short-day-grown *Arabidopsis* plants to long-day conditions, causing essentially photoperiod stress by 8 h light prolongation, resulted in an improved resistance against *B. cinerea* (Cagnola et al., 2018). Prolonged light exposure due to transfer from short to long days resulted in lower nuclear abundance of CONSTITUTIVE PHOTOMORPHOGENIC1 (COP1), thereby leading to stabilization of DELLA proteins and increased expression of the JA-signaling gene *JASMONATE INSENSITIVE1* (*MYC2*) (Cagnola et al., 2018). Exposure of maize to a prolonged light period increased resistance against *C. carbonum* race 1 (Marla et al., 2018). These studies indicate that the response to photoperiod stress is conserved among different plant species and that it has similar effects on pathogen resistance. Future research needs to identify the specific mechanisms conferring the improved pathogen resistance in photoperiod stress-treated plants.

## Conclusion and Future Research Perspectives

The photoperiod provides plants with information to synchronize their developmental program with the prevailing season. It is used to match the optimal conditions for offspring production and to alleviate the threats of seasonal stresses occurring at the same time every year. In this review, we have summarized the complex photoperiod sensing mechanisms (Figures 1, 2) and especially focussed on the role of the photoperiod in plant responses to cold, drought, osmotic and biotic stresses (Figure 3 and Table 1). While the molecular mechanisms of photoperiod-dependent regulation of cold, drought, and osmotic stress are at least partly elucidated, the impact of the photoperiod on biotic stress responses remains descriptive.

Recent studies (Nitschke et al., 2016, 2017) revealed that a sudden prolongation of the photoperiod causes a new form of abiotic stress, namely photoperiod stress, resulting in a nightly ROS accumulation in the apoplast and a stress response resembling pathogen infection (Abuelsoud et al., 2020; Cortleven et al., 2021). Photoperiod stress signals might have an adaptive value, for example by acting as a priming agent, which improves the plants’ performance to future stresses. The ecological relevance of photoperiod stress needs to be unveiled. It has been hypothesized that changes in intensity and ratios of wavelength during dawn and dusk that depend on weather conditions may modulate the output of the photoperiod sensing system (Abuelsoud et al., 2020), but the experimental proof is missing.

In view of the impact of the photoperiod on plant responses to pathogens, it may be envisaged that controlled changes of the photoperiod have a perspective for application in greenhouse farming. In these controlled environmental conditions the duration, intensity and wavelength composition of the illumination can be precisely regulated by light-emitting diodes (LEDs), which holds the potential to improve crop yield and quality (Jones, 2018; Lazzarin et al., 2021). LEDs can also be used for pest management. Several studies showed the influence of the light environment in greenhouses (including the length of the photoperiod and light quality) (for review, see Gallé et al., 2021; Lazzarin et al., 2021), on plant resilience against pathogens such as *B. cinerea* in strawberries (Meng et al., 2020) or tomatoes (Yang et al., 2015). Understanding how supplemental light through LEDs acts on plant growth and defense may lead to novel sustainable horticultural methods for pest management.

## Author Contributions

VR and AC wrote the draft of the manuscript. VR, AC, and TS revised the manuscript. All authors contributed to the article and approved the submitted version.

## Conflict of Interest

The authors declare that the research was conducted in the absence of any commercial or financial relationships that could be construed as a potential conflict of interest.

## Publisher’s Note

All claims expressed in this article are solely those of the authors and do not necessarily represent those of their affiliated organizations, or those of the publisher, the editors and the reviewers. Any product that may be evaluated in this article, or claim that may be made by its manufacturer, is not guaranteed or endorsed by the publisher.

## Footnotes

1
https://greennetwork.us.es/ATTRACTOR/
